# Which behaviour change techniques are associated with interventions that increase physical activity in pre-school children? A systematic review

**DOI:** 10.1186/s12889-023-16885-0

**Published:** 2023-10-16

**Authors:** Mosfer A. Al-walah, Michael Donnelly, Conor Cunningham, Neil Heron

**Affiliations:** 1 Centre for Public Health, School of Medicine, Dentistry and Biomedical Sciences, Queen’s University Belfast, Northern Ireland, UK; 2 Department of Physical Therapy, College of Applied Medical Sciences, Taif University, 21974 Taif, Saudi Arabia; 3 School of Health Science, Ulster University, Northern Ireland, UK; 4 School of Medicine, Keele University, England, UK

**Keywords:** Behaviour Change Techniques (BCTs), Template for Intervention Description and Replication (TIDieR) criteria, Young children (< 6 years old), Physical activity, Intervention

## Abstract

**Background:**

Insufficient physical activity (PA) is a significant risk factor that contributes to several health problems and there is a need to improve our understanding of how to increase PA, particularly among young children. This review (PROSPERO registration: CRD42022328841) investigated the relationship between behaviour change techniques (BCTs) and interventions that increased PA among pre-school children aged < 6 years old.

**Methods:**

Systematic searches of six databases were undertaken from inception to July 2022, updated in December 2022, to locate studies that evaluated interventions and reported a positive change in PA levels in children aged < 6 years old.

**Results:**

A total of 5,304 studies were screened, and 28 studies involving 10,605 subjects aged 2.5 to 5.9 years met the eligibility criteria. Each eligible study (*n* = 28) was independently appraised by two researchers using the Cochrane risk of bias tool. The BCT Taxonomy v1 and the Template for Intervention Description and Replication (TIDieR) guided the extraction and analysis of data, and this process led to the identification of 27 BCTs.

**Conclusions:**

Potentially promising BCTs for increasing PA among young children included ‘shaping knowledge,’ ‘antecedents,’ ‘goals and planning,’ and ‘comparison of behaviour.’ Future PA interventions that target young children should consider integrating these promising BCTs into their programmes. However, such consideration needs to be tempered by the fact that most of the reviewed studies were deemed to have a high or unclear risk of bias and/or were limited with respect to the populations that they targeted. Further research using rigorous methodologies is required to establish a higher standard that addresses the needs of young children who are expected to have insufficient levels of physical activity.

**Supplementary Information:**

The online version contains supplementary material available at 10.1186/s12889-023-16885-0.

## Introduction

Physical activity (PA) levels are an important indicator of obesity prevalence in early years and young childhood [1–3]. There is substantial literature to support the hypothesis that engagement in PA from birth to 5 years is associated with significantly improved health outcomes, not only in the short term but also over the life course of an individual [4, 5]. Higher PA levels are associated with better bone density, body composition, cardiovascular health, cognitive development, and motor skills [6, 7]. Behavioural patterns that emerge in early childhood have in turn been found to repeat through later childhood [8] and early adulthood [9]. However, while there is clear consensus in the literature regarding the benefits of PA in early childhood, evidence suggests that many young children are not active enough to derive these health benefits [10–12]. Several studies have shown that significant proportions of children do not meet the recommended PA levels of 180 min of light, moderate, and/or vigorous intensity PA (LMVPA) per day [13, 14]. A lack of physical activity can reduce energy expenditure while increasing calorie intake, resulting in excessive weight gain and obesity. In 2019, 38.2 million children under 5 were living with being overweight or obese and there was a paradigm shift from a prevalence in high-income countries to low- and middle-income countries as well [15]. Obesity in young children represents a pressing public health issue, which emphasises the need to target and reduce obesity-related habits and behaviours in early childhood [16–18]. Therefore, promoting physical activity among children and adolescents can be employed as a preventive measure against obesity and its complications through the provision of programmes to promote increased PA among young children [19, 20].

The focus of such programmes has generally been on the day care and/or the home environment [21]. Since 2010, several reviews have been published that have analysed findings from early childhood PA interventions [22–27] focusing on ECEC (Early Childhood Education and Care) settings, with mixed results reported. For instance, some studies showed that the participation of parents alongside children, and/or interventions that utilised a combination of structured (i.e., observed teacher or parent-led) and unstructured physical activities (for example, outdoor free play activities), demonstrated increased chances of success (i.e., improving PA levels in the target population) [24, 28]. Other studies have examined interventions implemented in the home and community environments [29–31]. A coordinated approach across environments can potentially yield greater impacts than single-setting efforts alone [32, 33].

There is a need for a comprehensive review of research on children's behaviour in various contexts, considering its complexity and interconnected effects [34], and given that what works in one setting may not work or not work as well in another setting [35]. Existing reviews lack information regarding the specific Behaviour Change Techniques (BCTs) used in interventions and their effectiveness in increasing physical activity (PA) levels among preschool children [36], as well as what particular BCTs produced the desired improvements in PA levels for preschool children. Globally, there is a paucity of research addressing this particular gap [37, 38].

A systematic review of existing research would help to identify the ‘active’ elements of interventions, alongside the factors which may effect change. The BCT Taxonomy v1 [39] provides a classification system through which the elements of an intervention, often referred to as the ‘active ingredients’ of interventions, can be identified and coded, aiding the precise evaluation and replication of effective behaviour modification methods. Researchers have analysed BCTs across various contexts, such as nutrition, postpartum smoking, and PA levels, to better understand interventions for improved health outcomes [40–42]. However, a recent review [43] of interventions targeting early childhood physical activity did not assess whether these interventions were based on theory, which components were focused on, and what behaviour change techniques (BCTs) were used to encourage positive changes in physical activity levels.

Addressing these research gaps is crucial for understanding intervention effectiveness across different settings. Utilising the 93-item BCT Taxonomy v1 [39] enables the identification of an intervention's 'active ingredients,' enhancing research quality, cost-effectiveness, and replicability. Additionally, the Template for Intervention Description and Replication (TIDieR) checklist [44] improves understanding of essential intervention elements and their potential for implementation into routine practice. Together, the TIDieR checklist and BCT Taxonomy offer a systematic way of identifying key intervention components and explaining the outcomes for a target population [45].

No systematic reviews currently exist which describe Behaviour Change Techniques (BCTs) and intervention theory in interventions to increase physical activity (PA) in young children. However, this systematic review aims to fill this gap by identifying and assessing BCTs and their effectiveness in promoting PA in young children by addressing two questions:

1. What are the most effective and commonly used BCTs in interventions for the promotion of PA in young children?
2. Which characteristics of interventions (manner of delivery, theoretical framework, intensity, dose, duration) are associated with their effectiveness?

## Methods

**Fig. 1:**
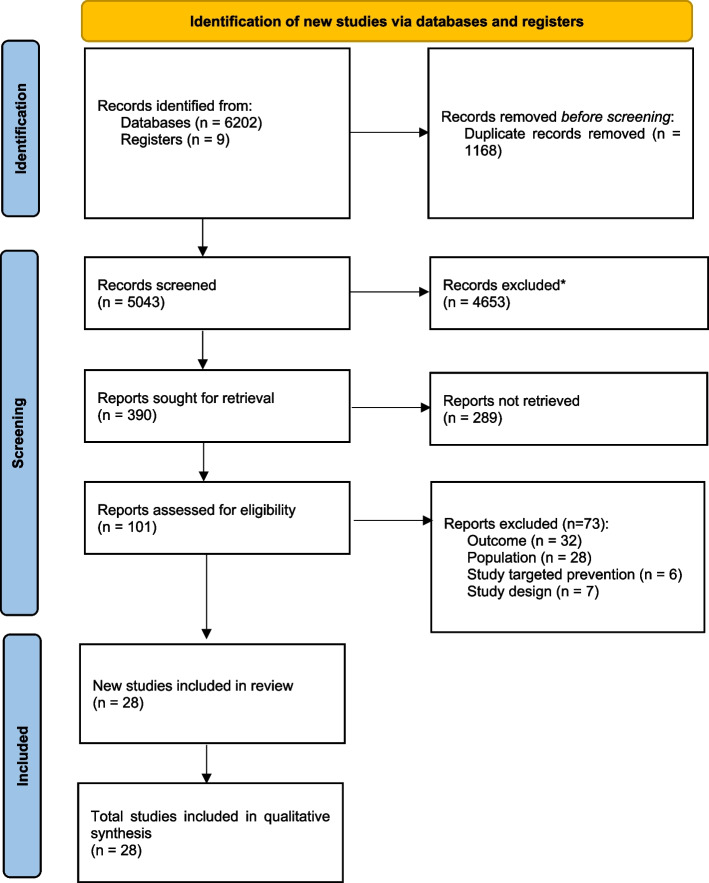
PRISMA Flow Chart. *No automation tools were used. *From: Page MJ, McKenzie JE, Bossuyt PM, Boutron I, Hoffmann TC, Mulrow CD, *et al*. The PRISMA 2020 statement: an updated guideline for reporting systematic reviews. BMJ 2021;372:n71. *https://doi.org/10.1136/bmj.n71

A systematic review was conducted following PRISMA guidelines [46] (see Fig. 1 and S1), and its protocol was registered with the International Prospective Register for Systematic Reviews (PROSPERO registration: CRD42022328841).

### Search strategy

Following previous similar reviews [22, 47] six databases were searched with the assistance of a specialised librarian: CINAHL, Ovid EMBASE, Ovid MEDLINE, PsycINFO, Web of Science, and Cochrane Library. For each relevant article, its respective reference list was also searched for additional potential studies. Further, reviewers attempted to locate unpublished and ongoing research by consulting experts in the field but did not uncover any additional eligible studies using this approach. The initial comprehensive search was carried out in July 2022 and was later updated in December 2022.

### Study selection and data extraction

The title, abstract, and discussion sections of articles were searched across the databases using medical subject headings (MeSH) keywords, and the population, intervention, comparator, outcome, and study design (PICOS) method was employed to specify inclusion criteria [48] (Table 1). Children were excluded from the study if they presented with any form of disability or diagnosed health conditions that significantly impacted on their ability to engage in physical activities.

**Table 1:** Keywords used in the search strategies and the selection criteria

**Concept**	**Search terms**	**Selection criteria**
**Population**	Infant*, baby, babies, toddler*, young, pre-school, early years, early childhood, young child*, child*	Children < 6 years old and not enrolled in a primary school at the time the intervention was delivered.
**Intervention**	physical education, BCT*, behaviour* change, behaviour change strategy*, behavioural change technique*, lifestyle change*, lifestyle intervention*, health promotion	Interventions aimed at improving PA levels among children < 6 years old. The intervention could take place in different contexts (e.g., community centres, clinics, hospitals, and private residences), use different modes of delivery (e.g., face-to-face, online, by text message, or phone call), over any duration.
**Comparison**		The review included studies that examined a PA intervention against a control group of children who received ‘usual care’.
**Outcome**	(Duration and type of participation in) Exercise, sport, physical fitness, walking, physically active*, MVPA, active transport, active play	Change in PA level assessed in terms of LPA, LMVPA, MVPA or CPM. Physical activity was reported as a total score or was scored across specific periods of the day (in hours/minutes or percentages).
**Study type**	Intervention* study, clinical trial, randomised controlled trial, controlled trial, RCT, cluster, quasi, experiment*	RCTs, Cluster-RCTs, and quasi*-*experimental studies with pre- and post-comparison, were included in the review if they were original articles that were peer-reviewed and published in English. Publication date and location were non-restrictive.

Two reviewers (M.A. and N.H.) conducted the screening of titles and abstracts, eliminating duplicates, and assessing relevance. Next, they individually obtained the full texts of relevant articles and used Rayyan software [49] for suitability analysis (Supplementary file: 2). Both reviewers (M.A. and N.H.) evaluated all included articles and, when needed, contacted the research author(s) to gather missing eligibility information. Any disagreements were resolved through discussions between the reviewers, with inclusion and exclusion reasons documented. In cases of unresolved disagreements, a third reviewer (M.D.) made the final decision.

Madden et al., [50] The review used the TIDieR checklist [44] to generate a description of the essential features of each intervention (Table 2). The following measures were adopted for this: duration (short ≤ 3 months, medium > 3 to ≤ 12 months, long > 12 months), number of sessions (low ≤ 10, medium > 10 to ≤ 20, high > 20), attrition (low ≤ 13%, medium > 13% to ≤ 26%, high > 26%), and adherence, as measured by previous authors [50, 51].

**Table 2:** TIDieR characteristics and intervention outcomes for individual studies

Study/year	Name	Why	What	What	Who	How	Where	How well?					
	Brief name	Rationale, theory, or goal or goal	Materials	Procedure	Provided	Mode of delivery		**Attrition**			**Adherence**		
								Low ≤ 13	Med > 13 ≤ 26	High > 26%	Low ≤ 30	Med > 30% ≤ 70%	High > 70%
Jones 2011 [52]	√	√	√	√	√	√	√			√			√
DeCraemer 2014 [53]	√	√	√	√	√	√	√	√					√
Pate 2016 [54]	√	√	√	√	√	√	√		√				√
Roth 2015 [55]	√	√	√	√	√	√	√		√			√	
Tucker 2017 [56]	√	√	√	√	√	√	√	√				√	
Alhassan 2012 [57]	√	√	√	√	√	√	√			√	√		
Annesi 2013 [58, 59]	√	√	√	√	√	√	√		-			-	
O'Dwyer 2012 [60]	√	√	√	√	√	√	√			√	√		
O'Dwyer 2013 [61]	√	√	√	√	√	√	√		-			-	
Palmer 2017 [62]	√	√	√	√	√	?	√		√			–	
Alhassan 2013 [63]	?	√	√	√	√	√	√		-			-	
Goldfield 2016 [64]	√	√	√	√	√	√	√			√		√	
Cardon 2009 [65]	?	√	√	√	√	√	√	√				-	
De Bock 2013 [66]	√	√	√	√	√	√	√			√			√
Jones 2016 [67]	√	√	√	√	√	√	√			√		-	
Brandes 2020 [68]	√	√	√	√	√	√	√		-			-	
Razak 2018 [69]	?	√	√	√	√	√	√			√			√
Adamo 2017 [70]	√	√	√	√	√	√	√		-			-	
Andersen 2020 [71]	√	√	?	?	√	√	√			√			√
Hoffman 2020 [72]	√	√	√	√	√	√	√		√			√	
LaRowe 2016 [73]	√	√	√	√	√	√	√		√			-	
Okely 2020 [74]	√	√	√	√	√	√	√			√		-	
Szpunar 2021 [75]	√	√	√	√	√	√	√			√		-	
Telford 2021 [76]	√	√	√	√	√	√	√		√				√
Wolfenden 2019 [77]	?	√	√	?	√	√	√			√			√
Segura 2021 [78]	√	√	√	√	√	√	√		-			-	
Finch 2014 [79]	√	√	√	√	√	√	√			√		√	
DeCraemer 2017 [80]	√	√	√	√	√	√	√		√			-	

Description of item (✓) = clear; (-) = minimal or no description; (?) = unclear description

Two reviewers (M.A. and C.C.) coded BCTs according to the BCT Taxonomy v1 (BCTTv1) [39]. This 93-item coding framework was used to guide researchers to independently identify and code BCTs that were present in the included studies that measured PA as a primary outcome. Only BCTs that were present in the intervention group and not in the comparator or control group were included [81]. As advised by Michie et al. [39], BCTs were coded as present beyond all reasonable doubt (+ +), present in all probability ( +), or absent (-). Beyond all reasonable doubt (+ +) was assigned if authors of a study presented evidence proving that a given BCT was applied to the target population and behaviour and explained how the BCT was utilised to enhance PA. Disagreements were resolved through discussions between coders and, if they remained unresolved, by consulting a third expert coder (N.H).

The study highlighted the contribution of each Behaviour Change Technique (BCT) to an intervention and its impact on the physical activity (PA) levels of the target population. This information was synthesised narratively and assessed based on criteria established in previous reviews [82–84]. Interventions were categorised into three levels of 'promise' based on their likelihood of improving outcomes compared to baseline:

- 1. Very promising: Significantly better outcomes in the intervention group compared to the control group (between groups).
- 2. Quite promising: Significantly improved outcomes within the intervention group (within groups), such as in pre-and post-test designs without a control group.
- 3. Non-promising: No statistically significant improvements in outcomes favouring the intervention group compared to the control group.

These categorisations helped evaluate the effectiveness of the interventions.

The potential for BCTs within interventions to change a given desired behaviour was measured through a ‘promise ratio’ for each BCT. This ratio was calculated by adding together all very- or quite-promising interventions that involved a specific BCT and then dividing this total by the number of non-promising interventions which featured that BCT. BCTs with at least twice as many promising (very or quite) as non-promising interventions (promise ratio of ≥ 2) were classified as promising [83]. BCTs with two or more promising interventions and no non-promising interventions (promise ratio of 0) were reported as indicating the number of promising interventions for which a given BCT featured.

#### Assessing the risk of bias

To assess the potential risk of bias, the Cochrane risk of bias method was employed [85]. The following factors were considered in the assessment of potential bias: the creation and disguising of distribution sequences; blinding of participants, personnel, and result assessors; availability of all relevant outcome data; the presence of selective reporting bias; and any other potential sources of bias such as financial conflicts. In turn, the potential for bias in each area was ranked as low, unclear, or high. Two researchers (M.A. and N.H.) performed the bias assessment. If they could not reach consensus through debate and discussion, a third researcher (M.D.) was consulted.

#### Synthesis and analysis

Study data was compiled and organised systematically. Following this, the TIDieR checklist and BCT Taxonomy v1 were utilised to identify and define essential intervention components, with the data then being presented in tabular form. A meta-analysis or meta-regression was deemed inappropriate for several reasons including studies having a high level of heterogeneity and varying in terms of intervention settings and components, a restricted number of studies available for each PA outcome, small sample sizes in a few studies, and a lack of comparability between the outcomes of different PA measures.

## Results

A total of 6,202 studies were identified via electronic searches, and nine studies were added after reviewing reference lists. After duplicates were omitted, 5,043 studies remained. The title and abstract of each paper were screened against eligibility criteria. This process left 101 potentially eligible publications. Following a full-text review, 73/101 were excluded. The remaining 28 studies were included in the review. A PRISMA flow chart outlining the identification of studies at each review stage is presented in Fig. 1.

### General characteristics of studies included in the review and as per TIDieR

The characteristics of the 28 included studies are presented in Table 2, which summarises the ‘intervention brief name,’ ‘why,’ ‘what,’ ‘who,’ ‘how,’ ‘where,’ along with ‘when and how much,’ while Table 3 details the full data extraction of TIDieR characteristics. Sixteen (57%) studies were found to be exclusively PA-focused, while the remainder considered multiple health behaviours and outcomes such as sedentary behaviour (SB) and/or BMI levels. Most of the 28 studies originated from the USA and Australia, comprising seven studies from each, followed by Canada (4), Britain (3), Germany (3), Belgium (2), and Norway (1). One study included data from six EU countries (Belgium, Bulgaria, Germany, Greece, Poland, and Spain). Most studies (*n* = 22) used a cluster RCT design. The number of childcare centres that participated in each study ranged from 2 to 43, with sample sizes ranging from 38 to 2438 participants. Seven studies recruited fewer than 100 participants. Multiple studies (*n* = 12) were conducted with children aged between three and five. Intervention duration across the included studies ranged from 4 weeks in two studies [63, 72] to 24 months [54].

**Table 3:** Ratio of BCTs to promise

**BCT label**	**Times** **used (*n* = 28)**	**(%)**	**Presence in very/quite** **promising interventions**	**Presence in non-promising interventions**	**Promise** **ratio**^**a**^
**1. Goal setting (behaviour)**	**4**	**14.2**	**3**	**1**	**3**
2. Goal setting (outcome)	1	3.6	1	0	1
3. Problem solving	5	17	2	3	0.66
**4. Action planning**	**14**	**50**	**10**	**4**	**2.5**
5. Monitoring of behaviour by others without feedback	2	7.14	1	1	1
6. Self-monitoring of behaviour	4	14.2	2	2	1
7. Self-monitoring of outcome(s) of behaviour	1	3.6	1	0	1
9. Feedback on outcome(s) of behaviour	4	14.2	2	2	1
10. Social support (unspecified)	3	10.7	1	2	0.5
**12. Instruction on how to perform the behaviour**	**15**	**53.5**	**12**	**5**	**2.4**
13. Information about health consequences	5	17	3	2	1.5
15. Demonstration of the behaviour	18	64.2	10	8	1.25
16. Prompts/cues	1	3.6	0	1	0
**17. Behavioural practice/rehearsal**	**7**	**25**	**5**	**2**	**2.5**
18. Habit formation	3	10.3	1	2	0.5
19. Graded tasks	2	7.14	1	1	1
20. Credible source	2	7.14	1	1	1
21. Non-specific reward	2	7.14	1	1	1
22. Social reward	1	3.6	0	1	0
23. Restructuring the physical environment	13	46.4	8	5	1.6
24. Restructuring the social environment	8	28.5	5	3	1.7
**25. Adding objects to the environment**	**16**	**57**	**11**	**5**	**2.2**
26. Identification of self as role model	2	3.6	0	2	0
27. Remove punishment	1	3.6	0	1	0

^a^Promise ratio denotes the number of very or quite promising interventions in which a behaviour change technique occurred divided by the number of non-promising interventions in which it featured. Rows in bold denote BCTs associated with a promise rate > 2 or used in promising interventions in at least two interventions

Regarding Materials and Procedures (What), apart from one study [71], all of the studies detailed the materials that were used for the interventions (e.g., newsletters, posters, music CDs, stickers, child achievement cards [52, 55, 57, 60, 61] equipment [52, 71, 72] and additional face-to-face support [79]. Except for one study [79], which used a pedometer, the remaining studies (*n* = 27) used accelerometers to assess PA, which were either waist-worn (*n* = 26) or wrist-worn in one study [68]. Most studies categorised PA using the ‘Pate’ [86] and ‘Sirard’ [87] cut-off reference points. Other methods of PA level measurement included pedometers (steps/day) [79, 80] and direct observation tools.

In general, for the studies that were conducted in an educational setting, educators and other staff received professional support to deliver the intervention objectives and components before the intervention commenced, although the intensity and frequency of provided training and resources varied from study to study. For example, O’Dwyer et al. [88] and Finch et al. [79] incorporated four to eight hours of staff training. Four studies involved parents [53, 64, 66, 88] (Table 2).

Specific intervention theories were specified in fourteen studies. The socioecological model was mentioned in five studies [54, 61, 70, 79, 88]. Two of the studies involved social cognitive theory alone [67, 74]; the other two studies incorporated social cognitive theory alongside either Self-Efficacy [58] or the Theory of Planned Behaviour [72]. The PRECEDE-PROCEDE model was used in two studies [53, 56], while general systems theory [89], communities of practice [71], and social development theory were each used in one study [76].

With regard to Intervention Facilitator Delivery to Children (Who), heterogeneity was evident regarding who delivered the interventions that led to the improved PA outcomes in the target population of young children. Most (68%, *n* = 19) interventions were facilitated by educators alone. Research staff/experts were responsible for the delivery of one intervention [60], while in five studies, the intervention was delivered by both researchers/experts and childcare staff [52, 61, 63, 75, 89]. Further, in one study, the intervention was overseen by a professional who offered training workshops to child healthcare practitioners, while another was exclusively conducted by external gym trainers [89]. In another, the intervention was carried out in cooperation with a peer coach who gradually introduced training components to instructors over a weekly period on-site [76].

Regarding the Intervention Mode of Delivery (How), all studies facilitated the intervention for the targeted population face-to-face except for one that was conducted via an online method [72]. In terms of location of intervention (Where), twenty-three studies focused exclusively on childcare settings. Five studies were undertaken in a childcare setting that incorporated a home component [53, 60, 70, 74, 89], whilst one was conducted online. The intervention included several components accessible from the WE PLAY website [72] (Table 2).

### Intervention duration and intensity (How long and how much), Adaptations (Tailoring and monitoring) and Attrition and adherence (How well)

Eleven interventions included in the review had a short duration (≤ 3 months) [56, 60–63, 65, 69, 72, 77, 78, 80], sixteen had a medium duration (> 3 to ≤ 12 months) [52, 54, 56, 60, 61, 66, 67, 70–72, 74, 76, 88, 89], and one had a longer duration (> 12 months) [54]. The average duration of the interventions in all of the included studies was 23.7 weeks. Twelve intervention groups incorporated a high number of sessions (> 20 sessions) [53–55, 57, 58, 64, 67, 68, 70, 73, 74, 89], ten had a medium number (> 10 to ≤ 20 sessions) [52, 60, 62, 63, 69, 71, 75–77, 79], and six had a low number (≤ 10 sessions) [56, 61, 65, 72, 78, 80]. Where required, interventions were tailored to participants’ ability and further adjusted where necessary. Most studies (*n* = 23) tailored interventions via personalised goal setting, a progressive review of weekly goals, problem solving, and individualisation of the frequency and intensity of the exercise component. No studies reported undertaking modifications. Five studies reported no intervention tailoring [60, 65, 67, 70, 77].

Across the studies reviewed, attrition rates (i.e., participants dropping out from the study) varied considerably. Three studies reported low attrition levels (≤ 13%) [53, 56, 65], six reported medium attrition levels (> 13% to ≤ 26%) [54, 55, 62, 72, 73, 76], while the remaining twelve reported high attrition levels (> 26%). Seven studies provided no information regarding attrition levels [58, 61, 63, 68, 70, 78, 80]. Adherence rates (i.e., participants who remained in the study but might not have completed the intervention components as required) also varied. Two studies reported a low adherence rate (≤ 30%) [57, 60], four studies reported medium rates (> 30% to ≤ 70%) [55, 56, 64, 71] and nine reported high adherence rates (> 70%) [52–54, 69, 71, 76, 77, 79, 89]. Finally, adherence rates were not reported in fourteen studies [58, 61–63, 65, 67, 68, 70, 72–75, 78, 80].

### Risk of bias

**Fig. 2:**
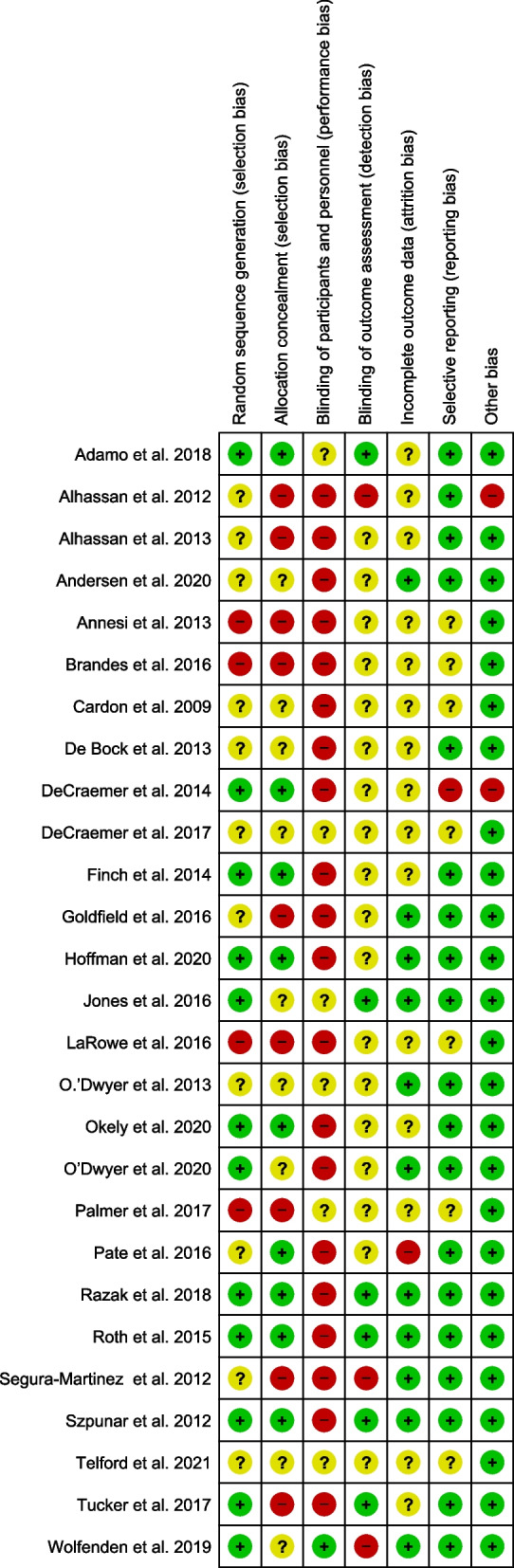
Risk of bias summary: assessment by review authors of each risk of bias item for the included studies

**Fig. 3:**
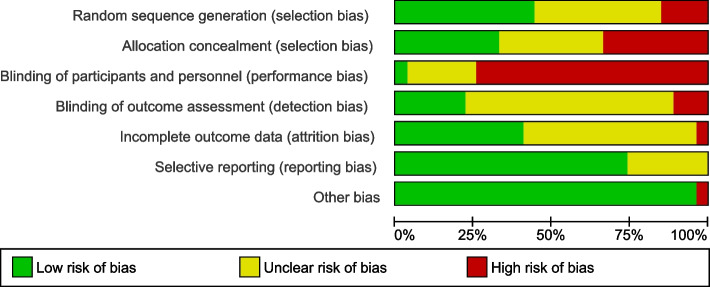
Risk of bias graph: results of assessment by review authors regarding each risk of bias item, presented as percentages across all included studies

Figures 2 and 3 summarise the risk of bias assessments and details about each risk of bias item, respectively. As insufficient information was provided, it was unclear whether random sequence generation was adequately performed in eleven studies [54, 57, 61, 63–65, 71, 76, 78, 80, 89]. Potential bias due to allocation sequence concealment was unclear in ten studies [60, 61, 65, 67, 70, 71, 76, 77, 80, 89]. Twenty-two studies were assessed as having a high risk of performance bias because they did not blind participants to the intervention. In five studies, the potential for bias was unclear due to insufficient information [61, 62, 67, 76, 80]. With respect to detection bias, six studies blinded outcome assessors [55, 56, 67, 69, 70, 78], and the potential risk for bias was low. Regarding attrition bias, fourteen studies offered insufficient information about the number of children who dropped out at the follow-up stage and the reasons for not continuing with the intervention program. One study [54] was deemed to be high risk because of the high dropout proportion (greater than 20%). Most studies (*n* = 20) provided sufficient information to assess the risk of selective reporting, and this risk was low, with one exception that did not adjust its analysis to factor in the effects of clustering [57]. Eight studies did not provide enough information to assess the risk of selective reporting [56, 58, 62, 65, 68, 73, 76, 80].

### PA Outcomes

#### PA Outcomes for RCTs

In 16 out of 28 interventions analysed, the personnel who were responsible for intervention delivery were encouraged to provide additional time for targeted children to undertake either structured or unstructured PA (Table 2). Participants were encouraged to undertake 20–60 min of additional PA, two to three times per week. A few studies undertook modifications to the indoor environment [75, 80], the outdoor environment [63, 69], or both the indoor and outdoor environments [77, 79]. However, the studies did not explicitly state if they targeted light intensity physical activity (LPA), moderate-to-vigorous physical activity (MVPA), or both. However, an examination of intervention strategies found that most targeted either MVPA or LPA and MVPA combined, rather than LPA by itself.

Fifteen studies (53%) reported significant changes in PA outcomes post-intervention. Significant changes in MVPA and VPA were recorded in eleven studies [53, 57, 58, 62, 63, 68, 69, 71, 73, 78, 89]. Seven of these interventions involved the provision of additional time for PA, while two studies included modifications to the environment [54, 57], and one study incorporated a combination of both additional time for PA and environmental modifications [66]. Two studies recorded significant changes in overall PA [56, 76], while two studies recorded significant changes in the number of steps [79, 88].

#### Outcomes as per TIDieR components

Core intervention characteristics linked with increases in PA levels among young children were recorded. Of the twenty-eight reviewed interventions in which young children participated, fifteen experienced PA improvements.

Regarding the interventions involving a named theory, 60% recorded an increase in the target population’s PA levels (*n* = 9), compared with 40% that did not reference a theory (*n* = 6). Eleven interventions were completed within three months, and 55% (*n* = 6/11) recorded an increase in young children’s PA levels (*n* = 6) [56, 61–63, 69, 78]. Sixteen interventions were delivered over a 3–12-month period; 59% (*n* = 9/16) reported an increase in PA [53, 57, 58, 65, 68, 71, 73, 76, 89]. One intervention took more than 12 months to deliver and found no significant increase in young children’s PA post-intervention [54]. Of the interventions that delivered a high number of sessions (21 +), 64% showed positive PA changes (*n* = 12). 59% of interventions with a medium number of sessions (> 10 to ≤ 20 sessions) showed positive PA outcomes, while 33% with a low number (≤ 10 sessions) showed positive PA outcomes.

### PA Outcomes according to BCTs

Only 27 out of 93 possible BCTs were used at least once in an included study, with an average of six BCTs used per study (range 3–10). The median number of BCTs used was split between ‘very/quite promising’ (*n* = 15) and ‘non-promising’ interventions (*n* = 13). A summary of the BCTs that were identified and coded in the 15 so-defined effective interventions is presented in Table 3. No single BCT was used across all interventions. The most frequently reported BCTs were ‘demonstration of the behaviour’ (*n* = 18, 64.2%), ‘adding objects to the environment’ (*n* = 16, 57%), ‘instruction on how to perform the behaviour’ (*n* = 15, 53%), and ‘action planning’ (*n* = 14, 50%). Five BCTs were assessed as promising (with a calculated promise ratio of ≥ 2): Goal setting (behaviour); Action planning; Instruction on how to perform the behaviour; Behavioural practice/rehearsal; and Adding objects to the environment. Two BCTs (‘self-monitoring of outcome(s) of behaviour and goal setting (outcome)’) featured in a ‘very/quite promising’ study only. Four BCTs featured only in ‘non-promising’ interventions (‘social reward,’ ‘prompts/cues,’ ‘identification of self as role model,’ and ‘remove punishment’). The ratios of intervention promise to BCTs ranged from 3 to 0.66 and are detailed in Table 3.

## Discussion

This systematic review is the first to comprehensively determine behaviour change theories and techniques used in interventions targeting physical activity (PA) in children under 6 years old, while also evaluating the interventions based on TIDieR guidelines. The review examined 28 studies that detailed these interventions. These studies included cluster randomized controlled trials (RCTs), and quasi-experimental designs, with varying levels of methodological quality, ranging from unclear to a high risk of bias. Given concerns about methodological quality, the findings from these studies should be interpreted cautiously.

The review found that interventions comprising multiple components, such as training, snack behaviours, physical education lessons, parental involvement, transitions, and challenging free play, were associated with better PA outcomes in young children. These interventions typically lasted between 3 and 12 months and involved multiple sessions per week.

Several Behaviour Change Techniques (BCTs) showed promise in these intervention studies, with the most promising being Goal setting (behaviour) (*n* = 4/28), Action planning (*n* = 14/28), Instruction on how to perform the behaviour (*n* = 15/28), Behavioural practice/rehearsal (*n* = 7/28), and Adding objects to the environment (*n* = 16/28).

The results of the reviewed studies indicated that approximately half of the interventions were informed by theory, mainly the social ecological model (*n* = 5/14) and social cognitive theory (*n* = 4/14). It is possible that theory was employed in the development and delivery of other interventions but was not reported in the papers that met the eligibility criteria. Furthermore, the interventions that incorporated theory into their planning and implementation were more effective in promoting increased physical activity among children (60%), in contrast to interventions that lacked such theories. Only 40% of interventions without theoretical foundations were successful in achieving the same outcomes. This finding is consistent with the conclusions of other systematic reviews [90, 91] which demonstrated that interventions that incorporated theory into their planning and implementation resulted in significant increases in physical activity among children.

In previous studies [64, 92], goal setting and action planning components were integral parts of the PA intervention. They employed a child-centred approach, allowing children to actively participate in the goal-setting process. At the beginning of the intervention, each child was encouraged to set personalised goals related to physical activity participation. The goals were specific, measurable, achievable, relevant, and time-bound (SMART) to promote clarity and facilitate progress monitoring. For example, a child might set a goal to engage in at least 30 min of moderate to vigorous physical activity (MVPA) five days a week [93].

Following goal setting, action planning was implemented to help children translate their goals into specific actions. This involved breaking the goals down into manageable steps and identifying potential barriers and strategies to overcome them. The children were guided by trained facilitators who provided support and helped them develop action plans. For instance, if a child's goal was to increase their daily MVPA, they would work with the facilitator to identify activities they enjoyed, such as riding a bike or playing soccer, and plan when and how they would engage in these activities [94].

To foster a sense of ownership and autonomy, children were encouraged to take the lead in setting their goals and action plans, with facilitators providing guidance, feedback, and motivational support throughout the process. Regular check-ins and discussions were conducted to assess progress, address challenges, and make any necessary adjustments to the goals and action plans [59].

By incorporating goal setting and action planning within the PA intervention, researchers aimed to empower young children to take an active role in shaping their physical activity behaviours. These BCTs have been shown to be effective in promoting behaviour change and fostering sustainable habits, even at a young age [59, 93].

Interventions must clearly articulate not only the theory used but also define how the chosen theory will guide the design, implementation, and evaluation of the intervention. Additionally, it should be acknowledged that motivational and environmental factors play a significant role in behaviour change and that researchers should consider the most appropriate approaches and theories for young children, such as family systems theory [78] and transactive goal dynamics theory [53], along with integrated approaches such as the behaviour change wheel [73] and intervention mapping [95], a six-step process that aims to improve health behaviours and environmental conditions considering the larger social and environmental context in which people live.

This review highlights that interventions designed to promote physical activity (PA) in young children often incorporate the use of specific Behaviour Change Techniques (BCTs) such as goal setting and action planning, which are tailored to the children's needs and abilities.

Goal setting involves defining a realistic objective that the child aims to achieve within a certain timeframe. In PA interventions, these goals typically relate to increasing the duration, frequency, or intensity of physical activity. The included studies employed various strategies for goal setting, including personalised goal setting and visual aids like charts, stickers, or progress trackers. These aids make goal setting more engaging and tangible for children.

Action planning, on the other hand, entails breaking these goals down into actionable steps or specific behaviours that children need to engage in to work towards achieving their goals. Practical strategies for implementing action planning include structured activity programmes [96] involving parents [97] and peers and using behavioural prompts, these prompts serve as cues to remind children to engage in planned physical activities and are effective in promoting PA [98].

Implementing these strategies enhances goal setting and action planning for young children in a practical and engaging way. Clear goals, visual aids, rewards, structured programmes, parental involvement, peer engagement, and behavioural prompts boost motivation and progress awareness, and support children in achieving physical activity goals. Further research on these strategies in PA interventions for young children could provide practical insights.

The review found that the most frequently recorded BCTs were ‘shaping skills’ (i.e., providing instruction on how to perform a behaviour) and ‘comparison of behaviour’ (i.e., a demonstration of how to perform a behaviour). These findings are not surprising given the nature of the interventions (i.e., group-based activity classes led by an expert practitioner) [99]. However, when analysing BCTs linked to promising interventions, a somewhat different pattern emerged. In over two-thirds of these interventions, extra health information and guidance were offered, and action planning included behavioural goal setting. Other significant BCTs included antecedents, present in at least 73% of promising interventions. Previous research has shown that combining self-regulation related BCTs (goal setting, problem-solving, and self-monitoring) yields better outcomes than using just one of these techniques [100].

Collectively, both adopting a theory to underpin an intervention and the specification of BCTs as active ingredients emerged as important indicators of success. Furthermore, intervention effectiveness was also found to be influenced by aspects such as who delivered the intervention, when and where it was delivered, and for what duration [83]. In assessing these factors, the TIDieR guidelines were used [44]. The person offering the intervention seems to affect outcomes differently. This was likewise highlighted by other reviews of PA interventions [101].

‘Who’ implements the intervention is a key intervention design aspect [102]. According to our review, the ideal type of intervention has not been determined; nevertheless, several studies have proven that when correctly educated, a range of providers may give successful health behaviour interventions [103, 104]. This systematic review found that researchers delivered more effective interventions than educators or other providers. Among interventions improving physical activity in young children, most (68%, *n* = 19) were conducted solely by educators. One was led by researchers/experts, and six involved both researchers/experts and childcare providers, with no consistent effectiveness pattern emerging based on the provider. Source credibility is vital in designing successful health promotion interventions and strategies. Previous research [105, 106] has shown that the sincerity of delivery or training may be more important than who is delivering the intervention. While training is important, it is also crucial to consider the credibility of the person delivering the intervention.

The person (for example, day care staff) who delivered the intervention seemingly had a limited impact on the outcome. This was also identified in other reviews of PA interventions [107]. Regarding the when and where, the interventions were mainly delivered in the childcare setting. Five were delivered outside of such settings, with mixed effects on PA behaviour being reported (i.e., four studies were conducted in the young/childcare setting but included a home component, while one was delivered online). Some studies have suggested that future interventions should embrace innovative and unconventional methods when being developed and implemented. A particularly interesting relevant example for children and adolescents, suggested by Benzing and Schmidt (2018), is exergaming, which refers to digital games that necessitate physical movements to be played. This creates an interactive gaming experience that provides a means of engaging in physical activity (PA) [108]. They highlighted the advantages of exergaming in promoting physical activity (PA) and health, including enhanced PA enjoyment, its suitability for specific populations (for example, children with attention deficit hyperactivity disorder), and the ability to customise experiences. However, they also noted drawbacks, including technical limitations and challenges in sustaining these programmes in the long run.

To provide directions for future research and practice in the promotion of PA and health through emerging technology, other suggested areas of research on innovative approaches to PA in children include exploring the benefits of applying mobile apps, wearable devices and social media, and investigating the application of augmented reality and virtual reality games in real-world settings [109].

By adopting this unique method, the intervention becomes more engaging, interactive, and tailored to an individual's preferences and needs. It leverages the power of technology and game-like elements to motivate and sustain behaviour changes over time. This approach might potentially result in improved intervention outcomes, such as increased adherence to physical activity, higher levels of enjoyment, and better long-term maintenance of exercise habits.

Most interventions took between 3–12 months, with 59% of these being positively associated with an increase in target population PA levels. This accords with the findings of a review on preadolescent PA interventions, where the greater effectiveness of interventions of between three and twelve months was also reported [19]. One study that lasted roughly two years had no positive impact on PA levels among preschoolers. This is possibly because of high dropout rates, as the study exhibited a high risk of attrition bias through twenty percent of dropouts. Furthermore, it was found that most interventions which positively changed PA levels involved at least two sessions per week. Little guidance currently exists regarding how many sessions or how much contact with intervention providers is necessary for PA behavioural changes to occur [107]. However, this review results can offer some guidance regarding this issue. The question of how well the intervention was delivered focused on attrition and adherence at intervention sessions. The results were mixed, with just 30% of the interventions recording adherence rates above 70%. Further, some studies did not seem to provide information regarding intervention fidelity. This was also noted in another recent review [110]. Moreover, a similar review suggested that studies had varying adherence levels, ranging from 44 to 95%, with differing definitions and an average attrition rate of 24 [111].

There may be a relationship between attrition and the length or intensity of the intervention, with participants being more likely to drop out when the intervention lasts for a longer period or is more intensive. However, this relationship is not consistent across the studies and may depend on the characteristics of the intervention and the participants themselves, especially given that most of these studies were conducted on adults. For example, a systematic review and meta-analysis of adherence to physical activity interventions for three chronic conditions (cancer, cardiovascular disease, and diabetes) found that adherence rates did not differ between clinic-based and home-based programmes, and that dropout rates were relatively low and consistent across the samples [112]. Another umbrella review of interventions to improve physical activity among socioeconomically disadvantaged groups found that interventions that were more intensive tended to be more effective, but also reported common methodological limitations such as a high probability of selection bias, low response rates, and high attrition [113]. Therefore, more high-quality studies are needed to determine the optimal duration and intensity of physical activity interventions for different population groups and health outcomes.

Without a clear assessment of intervention fidelity, it is not possible to determine the reasons why an intervention may (or may not) have worked. Research and studies can be conducted within a scientific framework which ensures that solutions to the problem of intervention fidelity can be found by following appropriate models that include intervention fidelity within their guidelines, implementation, and evaluation procedures, such as the MRC model [114].

Most reviewed studies were found to have a high risk of bias. Just over a quarter (28%) clearly reported allocation concealment, while sixteen (57%) were classed as high or unclear with respect to risk of bias because of missing data and how this was treated. No study had low risk with respect to intervention delivery involving nonblinded research personnel. These were assessed as being at high risk of performance bias due to the inability to blind participants to the intervention. This risk of bias assessment indicated that the review findings should be treated with caution. The review also highlighted that more rigorously designed and evaluated research investigating the effects of PA interventions in young children is needed. The review findings indicate a significant research and intervention gap concerning physical activity in this population.

According to our review, physical activity interventions that have been implemented thus far have primarily been conducted in developed nations, with a noticeable lack of research and studies on such interventions targeting young children in developing countries. Such a lack of comprehensive studies and initiatives in developing countries, Asian countries, African countries, and certain European countries, raises concerns about potential long-term effects on obesity rates and physical activity levels in these regions. This is particularly important given the shifting epidemiological paradigm whereby causes of morbidity and mortality, such as obesity, cardiovascular diseases, and cancer [115–119], which were classically seen as ‘first world’ diseases, are now becoming apparent in developing countries. With obesity being a significant public health concern across all segments of society, particularly in young children, this significant gap in the field must be addressed [120, 121].

Such gaps in the research have resulted in a lack of understanding regarding the barriers that need to be overcome to effectively implement physical activity interventions in these countries. Furthermore, it cannot be assumed that intervention studies from one region (for example, in the global north, where most of the studies were published), are readily applicable to other geographical and cultural settings, as regional barriers to increased levels of physical activity may differ [122]. To effectively address these issues, interventions must be tailored to reflect cultural and religious distinctions among populations in these nations, and socio-cognitive, cultural, and environmental factors need to be considered. Further, regular evaluation of the effectiveness of physical activity interventions in these diverse contexts is crucial. Collecting data and feedback enables researchers to gain insights into intervention outcomes and adapt their methods accordingly. This iterative approach also facilitates continuous improvement and enhances the likelihood of achieving meaningful results.

## Strengths, limitations, and implications for future research

### Strengths

This systematic review has some notable strengths. It is the first study to apply TIDieR guidelines to identify the key characteristics of interventions targeting young children’s PA levels. The review also highlighted how some aspects seem to be inadequately reported on, such as the fidelity of intervention delivery. Nevertheless, it is worth mentioning that fidelity may have been reported separately in a process evaluation paper rather than an effectiveness paper. Furthermore, BCTTv1 was employed to identify the active ingredients of the interventions. As this was the first systematic review to critically appraise and synthesise insights from the included studies, it will enrich the knowledge of researchers, clinicians, and the general public, as it helps in identifying how and why some interventions work while others fail. In turn, this will aid the designing of more effective future PA interventions for young children.

Future research should therefore consider the use of such guidelines and methodological tools in describing interventions, as well as increasing formative work with young children to help develop interventions that are feasible, acceptable, and implementable [110, 114].

### Limitations

Despite its perceived strengths, we found that this review has two main limitations. First, the strict inclusion criteria adopted raises the possibility that some relevant studies may have been missed by the review process, despite the efforts made to mitigate this issue (see Methods). Second, there were a significant number of reports (n = 289 out of 390) which were sought for retrieval but were excluded due to limited accessibility.

### Implications for future research

This review has made a unique contribution to the literature in that it augments existing knowledge regarding key intervention characteristics, alongside behaviour change theories and techniques used in PA interventions aimed at young children. The review demonstrates the importance of reflecting on what theories best underpin interventions. It also highlights the need to describe with more precision the process by which PA interventions are informed and tested by specified theories.

According to our findings, the social ecology model and social cognitive theory (SCT) were the most utilised theories. It is important to acknowledge the strengths and limitations of each theory, such as SCT's focus on learning and doing in a social setting with an emphasis on social influence [123]. However, one of the key limitations of SCT is the assumption that a change in the environment, such as adding a pedometer, will automatically lead to changes in behaviour without taking emotions and motivations into account [124]. In general, behaviour change techniques (BCTs) that align with SCT have been shown to have a positive impact on intention but not necessarily on actual behaviour change. This suggests that while certain aspects of SCT may be effective in increasing physical activity, the emotional and motivational components of the theory need to be addressed to achieve maximum benefits [125].

To create successful physical activity interventions, BCTs should be used effectively by considering the target population and delivery. Future studies should employ a step-by-step approach, considering age and using structured BCTs, while outlining processes such as intensity, frequency, and delivery. They should also measure implementation fidelity and consider implementation factors, assess social cognitive indicators to gauge BCT impact, and provide precise details on BCT integration across contexts, especially considering potential future pandemics. Parental influence on a child's behaviour is another crucial factor. Children perceive parents as role models and internalise their actions, attitudes, and values, impacting on behavioural development.

Active parent involvement, including play, academic, and extracurricular activities, strengthens parent–child bonds and encourages positive behaviour. Supportive and loving environments further foster positive behaviours. However, a child's behaviour is also influenced by genetics, peers, and the social environment, so parents should create a nurturing atmosphere while acknowledging these factors.

Interventions should be evidence-based, comprehensive, and tailored to individual needs. A holistic approach addressing psychosocial factors and behaviour changes could lead to sustained changes and clinical benefits, benefiting society.

This review has shown that the interventions examined exhibited varying durations including short (< 3 months), medium (3–12 months), and long (> 12 months). The results revealed that the interventions lasting between 3–12 months showed a significant positive association (59%) with increased levels of physical activity (PA) in the target population. This observation is consistent with conclusions from a review of preadolescent PA interventions, which also indicated greater efficacy over the 3–12-month time frame [126]. Conversely, interventions lasting less than three months showed a lower (39%) positive association with increased PA levels among young children. A specific two-year study had no significant positive effect on PA levels in preschoolers, likely attributable to high dropout rates and the consequent risk of attrition bias, with approximately 20% of participants dropping out. This underscores the importance of addressing attrition bias in future studies. Understandably, this relatively long duration may seem extensive and might not be applicable across all relevant settings. The review also recommends incorporating a suite of behaviour change techniques (BCTs) that correspond with the chosen theory. Incorporating some form of BCTs, such as ‘goal setting (behaviour),’ ‘action planning,’ ‘instructions on how to perform the behaviour,’ ‘behavioural practice or rehearsal,’ and ‘adding objects to the environment,’ were found to correlate with PA level increases in the target population.

## Conclusions

This review provides a valuable starting point for developing future interventions to promote physical activity in young children under 6 years old. It pioneers the use of TIDieR guidelines and BCTTv1 to systematically evaluate and gain a better understanding of the key components targeting physical activity interventions for this age group. The review recommends incorporating behaviour change techniques that align with the underlying theory of the intervention. However, the findings should be approached cautiously due to the high risk of bias in the reviewed studies. Nevertheless, this review offers a valuable foundation for future research, emphasising the need for evidence-based, empirically grounded studies, particularly in regions lacking such interventions. Customising interventions to cultural contexts is also essential, drawing from international models and successful practices. Ultimately, this review's insights can guide the creation of effective, culturally relevant PA interventions for young children, aiding policymakers in addressing childhood obesity and sedentary behaviour challenges in communities.

### Supplementary Information

**Additional file 1.**

**Additional file 2.**

**Additional file 3.**

**Additional file 4.**

## Data Availability

All data generated or analysed during this study is included in this published article and its supplementary information files.
