# Supplementary material for: Which behaviour change techniques are associated with interventions that increase physical activity in pre-school children? A systematic review
Source: BMC Public Health. 2023 Oct 16;23:2013. doi: 10.1186/s12889-023-16885-0 (PMC10580560; doi:10.1186/s12889-023-16885-0)
Supplement: Supplementary file 1 — Additional file 1. [file 12889_2023_16885_MOESM1_ESM.docx]

**Example (MEDLINE) search strategy**

Database: Ovid MEDLINE(R) without Revisions <1946 to July Week 2 2022>

Search Strategy:

1 physical education/14045

2 BCT.mp.2845

3 behaviour change.mp. or behavior change/7132

4 behaviour change strategy.mp.37

5 behavioural change technique.mp.6

6 lifestyle change.mp. or lifestyle modification/2101

7 lifestyle intervention.mp.5673

8 health promotion/80335

9 Intervention.mp. or intervention study/1284792

10 clinical trial/537496

11 randomized controlled trial/582703

12 primary prevention/19954

13 RCTs.mp.48593

14 cluster.mp.271002

15 experiment/0

16 quasi experimental study/ or quasi.mp.63235

17 exercise/137123

18 sport/33831

19 fitness/ 0

20 walking/39996

21 physical activity.mp. or exp physical activity/321501

22 MVPA.mp.6030

23 active transport/31415

24 play/ or active play.mp.9474

25 infant/ 858697

26 baby/0

27 babies.mp.41110

28 toddler/0

29 preschool child/ or preschool.mp.994270

30 pre-school.mp.5274

31 early years.mp.4666

32 early childhood.mp.32546

33 young child.mp. 4759

34 exp child/2116179

35 9 or 10 or 11 or 12 or 13 or 14 or 15 or 16/1915348

36 17 or 18 or 19 or 20 or 21 or 22 or 23 or 24/387770

37 25 or 26 or 27 or 28 or 29 or 30 or 31 or 32 or 33 or/342427682

38 1 or 2 or 3 or 4 or 5 or 6 or 7 or 8/109305

39 35 and 36 and 37 and 38/2390

40 limit 39 to (human and english language and child <unspecified age>)/2324

41 limit 39 to (human and english language and child <unspecified age>)/2324
